# Uteroglobin, a Possible Ligand of the Lipoxin Receptor Inhibits Serum Amyloid A-Driven Inflammation

**DOI:** 10.1155/2014/876395

**Published:** 2014-03-23

**Authors:** Giovanni Antico, Monica Aloman, Katja Lakota, Lucio Miele, Stefano Fiore, Snezna Sodin-Semrl

**Affiliations:** ^1^Astellas Pharma US, Northbrook, IL 60062, USA; ^2^Advocate Christ Medical Center & Hope Children's Hospital, 4440 W 95th Street Oak Lawn, IL 60453, USA; ^3^Department of Rheumatology, University Medical Center-Ljubljana, 1000 Ljubljana, Slovenia; ^4^Departments of Medicine, Pharmacology, Biochemistry and Radiation Oncology, The University of Mississippi Medical Center, Cancer Institute, Jackson, MS 39216, USA; ^5^Sanofi-Aventis, Bridgewater Township, NJ 08807, USA; ^6^Faculty of Mathematics, Natural Science and Information Technology, University of Primorska, 6000 Koper, Slovenia

## Abstract

Serum amyloid A (SAA) production is increased by inflamed arthritic synovial tissue, where it acts as a cytokine/chemoattractant for inflammatory and immune cells and as an inducer of matrix degrading enzymes. SAA has been shown to bind lipoxin A_4_ receptor, a member of the formyl-peptide related 2 G-protein coupled receptor family (ALX) and elicit proinflammatory activities in human primary fibroblast-like synoviocytes (FLS). We report on the identification of uteroglobin, a small globular protein with potent anti-inflammatory activities, as a possible ligand of ALX. Uteroglobin-specific association with ALX was demonstrated by an enzyme immunoassay experiment employing a cell line engineered to express the human ALX receptor. Uteroglobin's interaction with ALX resulted in the inhibition of SAA responses, such as attenuation of phospholipase A_2_ activation and cellular chemotaxis. In FLS, uteroglobin showed an antagonism against SAA-induced interleukin-8 release and decreased cell migration. These novel roles described for uteroglobin via ALX may help elucidate genetic and clinical observations indicating that a polymorphism in the uteroglobin promoter is linked to disease outcome, specifically prediction of bone erosion in patients with rheumatoid arthritis or severity of IgA glomerulonephritis and sarcoidosis.

## 1. Introduction

Serum amyloid A (SAA) is an acute phase protein and inflammatory marker of rheumatoid arthritis (RA) and its disease progression [1]. During the acute phase response, SAA levels can increase up to 1000-fold in the circulation of humans, making it a major acute phase reactant (APR) [2]. SAA promotes the release of cytokines and chemokines in fibroblast-like synoviocytes (FLS) by binding to its receptors, such as receptor for advanced glycation end-products [3] or through lipoxin A_4_ receptor (ALX), also called formyl-peptide related 2 receptor [4].

ALX was originally reported to be present in FLS by our group in 2000 [5]. It is a member of the seven transmembrane G-protein coupled receptor family with different classes of ligands. Proinflammatory activation of cells expressing ALX was shown with bacterial and viral peptides, endogenous acute phase molecules, such as SAA, and the fibrinolytic receptor for urokinase [6–9]. An anti-inflammatory cascade of events was initially reported to be triggered by lipid ligands, such as endogenous arachidonate-derived lipoxygenase product lipoxin A_4_ (LXA_4_) [10–12], LXA_4_-derived mimetics, and epi-LXA_4_. However, the addition of annexin-1 (a glucocorticoid-induced anti-inflammatory protein) to the growing family of ALX ligands has provided evidence that this receptor can also modulate anti-inflammatory signaling [13] via proteins.

Uteroglobin (UG), a steroid-inducible, 16 kDa secreted protein with potent anti-inflammatory/immunomodulatory activities, was found to share short peptide sequence similarities with annexin-1, termed the antiflammin motif [14–17]. Endogenous nonapeptides or antiflammins were reported to carry regulatory effects on inflammation and overlapping biological activities [18–21]. UG and its peptide derivatives, the antiflammins, have also been reported to modulate effects on phospholipase A_2_ (PLA_2_) activity, phagocyte activation, formyl-methionyl-leucyl-phenylalanine (fMLP)- and complement fragment C5-induced chemotaxis, and phagocytosis of monocytes and neutrophils [22–25]. In contrast data has been reported on the ability of antiflammins to inhibit PLA_2_ activity [14, 19, 26, 27]. Even though the basic nonapeptide antiflammin-2 was demonstrated to be devoid of PLA2 inhibitory activity [28], Kamal et al. reported on its selective binding to human ALX, thus providing a potential mechanism for its anti-inflammatory properties [29].

Like annexin-1, UG is also known as a secretory, glucocorticoid-inducible protein [17], while displaying powerful immunomodulatory properties, similar to those of LXA_4_, such as inhibition of IL-6 release [30], reduction of cellular infiltrates in the airways [31], decreased synthesis of prostaglandins (namely, PGF_2_ and PGE_2_ [32]), and reduced lysophosphatidic acid plasma levels [33].

The ability of UG to limit the availability of intracellular arachidonate (by strongly inhibiting phospholipase A_2_ activity) and prevent the generation of lipid mediators has been proposed as the main mechanism leading to its anti-inflammatory activity [14, 15]. This has led us to investigate whether UG can also interact and signal via ALX, as well as elicit similar anti-inflammatory functions.

The current report indicates that UG interacts with ALX and, similarly to LXA_4_ and/or annexin-1, activates a potent negative feedback mechanism on cellular inflammatory events. We show that several previously reported UG biological responses could be based on its molecular interaction with ALX.

## 2. Materials and Methods

### 2.1. Ethics Statement

This study was performed in accordance with US guidelines and was approved by the Internal Review Board at the University of Illinois at Chicago, where the study was conducted.

### 2.2. Cell Cultures

Human primary synovial fibroblasts (FLS) obtained from arthroscopic knee biopsies were cultured in EMEM supplemented with 10% fetal bovine serum (FBS). Fresh medium containing 5% FBS was added to cell culture 24 hours prior to each experiment. FLS passages 7–11 were used.

The promyelocytic leukemia cell line (HL-60) was grown [34] in RPMI 1640 containing 10% FBS. HL-60 cells stably transfected with either empty pcDNA-3 (HL60^pcDNA3^) or pcDNA-3 containing the ALX open reading frame (HL60^ALX^) were cultured in presence of 400 *μ*g/mL of G418.

Chinese hamster ovary (CHO) cells were cultured in *α*-MEM supplemented with adenosine, deoxyadenosine, thymidine (0.2 mg/mL each), and 10% FBS. CHO cells stably transfected with either empty pINF plasmid (CHO^pINF^) or pINF containing the ALX open reading frame (CHO^ALX^) were selected in presence of 5 *μ*g/mL of puromycin.

Human endometrial cells-1A (HEC-1A) and HEC-1A^UG^ were kindly provided by Dr. Peri A. (Department of Clinical Physiopathology, University of Florence, Italy) and grown in McCoys medium, supplemented with 10% FBS and sodium bicarbonate (2.2 g/L). G418 was used as selective antibiotic for HEC-1A^UG^. Human recombinant UG (hrUG) was kindly provided by Claragen Inc. (Rockville, MD, USA). Human recombinant SAA1/2 (hrSAA) was purchased from Peprotech (Rocky Hill, NJ, USA).

### 2.3. RNA Isolation and Reverse Transcription-PCR

Total RNA was extracted with TRIZOL Reagent (Life Technologies, Rockville, MD). RNA reverse transcription (RT) system was used according to the manufacturer's instructions (Promega, Madison, WI, USA) and RT-PCR was performed as follows: for UG, forward primer (within exon 1): 5′-CTC ACC CTG GTC ACA CTG G-3′; reverse primer (within exon 3): 5′-TTG AAG AGA GCA AGG CTG GT-3′ at an annealing temperature of 50°C with 30 cycle amplification in order to obtain a 344 bp fragment. Nested reverse transcription-PCR was required for detection of UG in FLS, with the forward primer as stated above and the reverse primer (within exon 2): 5′-GGG TGT CCA CCA GCT TCT TC-3′ at an annealing temperature of 47°C with 30 cycle amplification to obtain a fragment of 180 bp in size. ALX analysis was conducted as previously published [5].

### 2.4. Antisense Oligonucleotides

Phosphorothioate oligos were purchased from IDT (Coralville, IA, USA). For ALX, previously published antisense and sense oligonucleotides were used [12]. For UG, the antisense oligo was 5′-C*T*G* AAA AGT TCC ATG GC*A* G*-3′ and the scrambled oligo was: 5′-C*C*G* TGC TGT ACC TAG TG*A*G*-3′ (∗ indicate phosphorothioate bonds). Oligos were added to cell culture supernatants at a final concentration of 8 *μ*g/mL for 48 h and readministered a second time for another 72 h prior to treatments.

### 2.5. Modified Enzyme Immunoassay (EIA)

CHO cells were cultured in 96-well plates up to 80% of confluency. The cells were then washed three times with PBS prior incubation with increasing concentrations of UG diluted in *α*-MEM (200 *μ*L/well) for 10 min at 4°C. After washing with PBS, the cells were incubated for 1 h at room temperature with goat anti-UG antibody (20 *μ*g/mL) diluted in a solution containing CHO^pINF^ culture supernatant and fresh *α*-MEM (5% v/v). The cells were then washed twice with PBS and incubated for 45 min at room temperature with 1 : 2000 dilution of horseradish peroxidase-conjugated anti-goat antibody (Vector laboratories, Burlingame, CA). Contribution of endoperoxidase was abolished by cell treatment with 0.01% hydrogen peroxide for 30 sec. After two washings with PBS, the cells were incubated at room temperature with o-phenylenediamine dihydrochloride solution in citrate buffer (Pierce Biotechnology, Rockford, IL) containing 0.03% hydrogen peroxide. The colorimetric reaction was stopped using 2 N sulfuric acid. Supernatants (200 *μ*L) from each well were transferred to a 96-well plate and color intensity measured at 490 nm. The values were normalized by protein content. Cells used in the assay were fixed in 10% trichloroacetate solution (1 h at 20°C). After drying, the content of each well was stained with 1% sulforhodamine B (Sigma, St. Louis, MO), washed in acetic acid and resuspended in 50% Tris base solution, followed by detection at 510 nm.

### 2.6. Phospholipase A_**2**_ (PLA_**2**_) Assay

Phospholipase activity was measured by determining the release of the free acid form of tritiated arachidonic acid after esterification of the labeled arachidonate to membrane phospholipids [35]. Values for tritiated AA release recovered in the supernatant were determined by *γ*-scintillation counting at indicated time points.

### 2.7. Interleukin-8 Release

ELISA was used to measure released IL-8 levels in culture supernatants as previously reported [5] and following manufacturer's instructions (Invitrogen, USA).

### 2.8. Chemotaxis Assays

The chemotaxis assays were performed as previously described [8, 16]. Briefly, HL-60^pcDNA3^ and HL-60^ALX^ were cultured overnight in RPMI containing 0.1% FBS and then seeded onto 8 *μ*m pore polycarbonate membrane transwells. SAA and UG were added to the lower chambers of the transwells at indicated concentrations. After 1 h incubation at 37°C, suspensions in the lower chambers were collected. Subsequently, the cells were treated with propidium iodide and analyzed for number and morphology by FACS.

Human FLS were plated to confluency onto transwells with 8 *μ*m pore polycarbonate inserts using either PC-1 or phenol red-free DMEM. After 12 h in serum-free conditions, chemotaxis was assessed for 2 h after addition of agonists into the lower chamber. Migrated cells were manually counted (Crystal Violet staining) or measured by metabolic colorimetric assay (formazan, EZ4U, ALPCO Diagnostics, Windham, NH, USA).

### 2.9. Statistical Analysis

All experiments were repeated at least three times (unless otherwise noted) and the mRNA results by RT-PCR were shown as a representative gel of two performed. Data are reported as mean ± SD. Means were compared between treated and control groups with Student's *t*-test and significant *P* values less than 0.05 determined to be statistically significant.

## 3. Results

### 3.1. Characterization of UG Interactions with ALX

**Figure 1:**
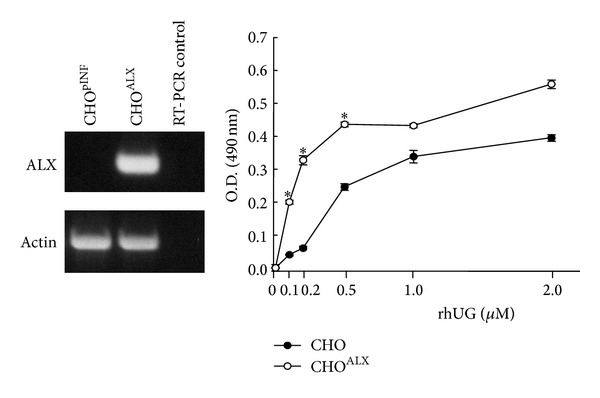
Exogenous hrUG binding by CHO cells expressing ALX shown by modified enzyme immunoassay. CHO cells expressing either the empty vector pcDNA3 vector (CHO^pINF^) or containing the full ALX open reading frame (CHO^ALX^) were exposed to increasing hrUG concentrations. The asterix notation indicates data points with a *P* value of <0.02 when comparing CHO^ALX^ to CHO^pINF^ data series. Results are plotted as a mean ± SD of quadruplicate determinations performed in three separate experiments. Reverse transcription-PCR was used to monitor the ALX mRNA expression in CHO cells stably transfected with ALX cDNA (inset). Control represents no template added to reaction. ALX is lipoxin A_4_ receptor or FPR2/ALX. The result is from a representative gel of two performed.

In order to determine whether UG can bind to ALX, we first used an adherent cell line Chinese hamster ovary cells (CHO), stably transfected with mock pINF vector as well as overexpressing the human ALX in a modified enzyme immunoassay (EIA). A higher and specific UG binding was observed in CHO^ALX^ cells as compared to mock CHO^pINF^ (Figure 1) with ALX mRNA expression only in the CHO^ALX^ transfected cells (Figure 1 inset).

The protein-receptor assays on the cells were performed at 4°C, since the interaction itself could be modified by cell activities, such as internalization of the protein/receptor complex, expulsion, or masking by intervening membrane events.

Since the UG-dependent dose curve in CHO^pINF^ indicates the existence of other proteins/receptors dependent on UG in these cells, such as fibronectin [36], we cannot exclude that further effects of UG go through alternative pathways.

### 3.2. Human Recombinant UG Modulates SAA-Induced ALX-Dependent Cell Signaling

**Figure 2:**
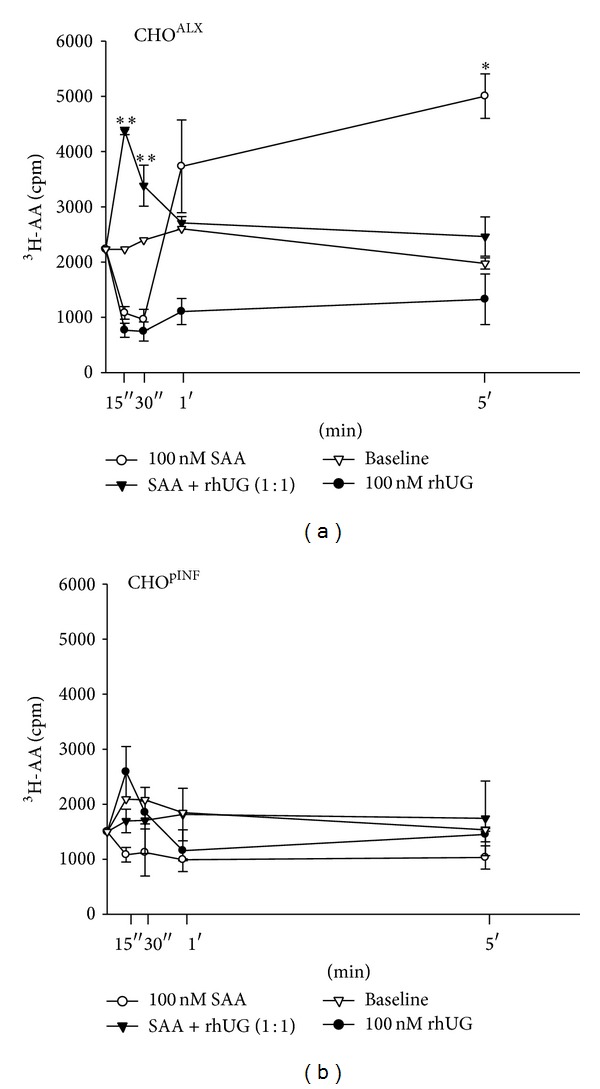
SAA-induced phospholipase A_2_ activity is attenuated by UG in CHO cells overexpressing ALX. Time response of SAA-induced PLA_2_ activity in CHO^ALX^ cells (a) over a 5-minute monitoring period in the presence and absence of UG, as compared to mock transfected CHO cells (b). Significance is noted by Student's *t*-test of SAA treatment versus SAA + UG treatment. ∗ for *P* < 0.005 and ∗∗ for *P* < 0.001. Results represent the mean ± SD of four separate experiments with triplicate determinations.

UG ability to modulate ALX signaling was determined in CHO cells by comparing the effects of UG and SAA, alone or in combination, on PLA_2_ activity (Figure 2). SAA upregulated PLA_2_ activation selectively in cells overexpressing ALX (CHO^ALX^, Figure 2(a)), while no response was obtained in control cells (CHO^pINF^, Figure 2(b)). This is in striking contrast to UG effects resulting in a selective downmodulation of basal levels of PLA_2_ activity (Figure 2(a)). Interestingly, results indicate that UG triggers an early peak of transient activity in the presence of SAA in CHO^ALX^. This early PLA_2_ activity peak is followed by a sustained inhibition of SAA-induced activation at later time points (Figure 2(a)). There is also a transient early peak of UG in mock transfected CHO cells (Figure 2(b)), which is lower than the early combined effect of SAA and UG in CHO^ALX^.

### 3.3. Functional Consequence of UG on SAA-Induced Release of IL-8 and Cellular Chemotaxis in Human Cell Lines and Primary Synovial Fibroblasts

**Figure 3:**
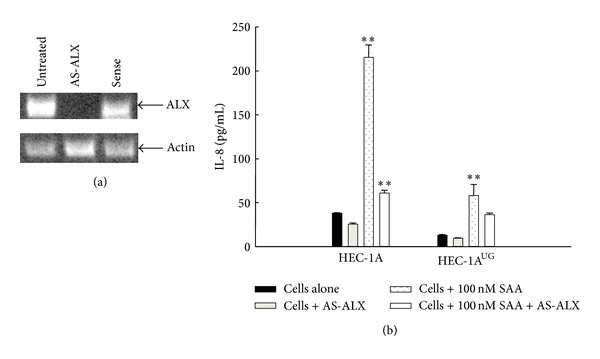
SAA-induced IL-8 levels were significantly downregulated in HEC-1A cells overexpressing UG. (a) Reverse transcription-PCR of ALX mRNA in HEC-1A cells and ALX-antisense (AS-ALX) oligo-treated HEC-1A cells. The result is from a representative gel of two performed. (b) SAA-dependent IL-8 release as measured by ELISA, in HEC-1A cells, is inhibited when pretreated with ALX-antisense oligo. In HEC-1A^UG^ cells SAA-induced IL-8 release is significantly lower in comparison to HEC-1A. Student's *t*-test for SAA versus AS-ALX treated HEC-1A cells or HEC-1A^UG^ cells was calculated, *P* < 0.0001 (∗∗). Data were normalized using number of cells. Results are the mean ± SD of three separate experiments with quadruplicate determinations.

Since endogenous synthesis of UG could contribute potential confounding elements, HEC-1A cells, which naturally do not express the UG gene, were used in comparison with HEC-1A engineered to overexpress UG (HEC-1A^UG^). Additionally an antisense oligo (AS-ALX) treatment was established to modulate the receptor expression in HEC-1A (Figure 3(a)). As shown in Figure 3(b), left side of the graph, wild type HEC-1A cells lacking synthesis of endogenous UG, responded to SAA with a fivefold increase in synthesis of IL-8 over the control, and yet released IL-8 levels were reduced to background level, when cell expression of ALX had been silenced by treating cells with AS-ALX. In HEC-1A^UG^ cells with endogenous UG synthesis, SAA-induced IL-8 released levels were also decreased (Figure 3(b), right side of the graph). UG secretion alone also decreased the baseline value of released IL-8 levels. The overall conclusion is that the SAA effect on IL-8 is still seen with UG present; however, is significantly attenuated.

**Figure 4:**
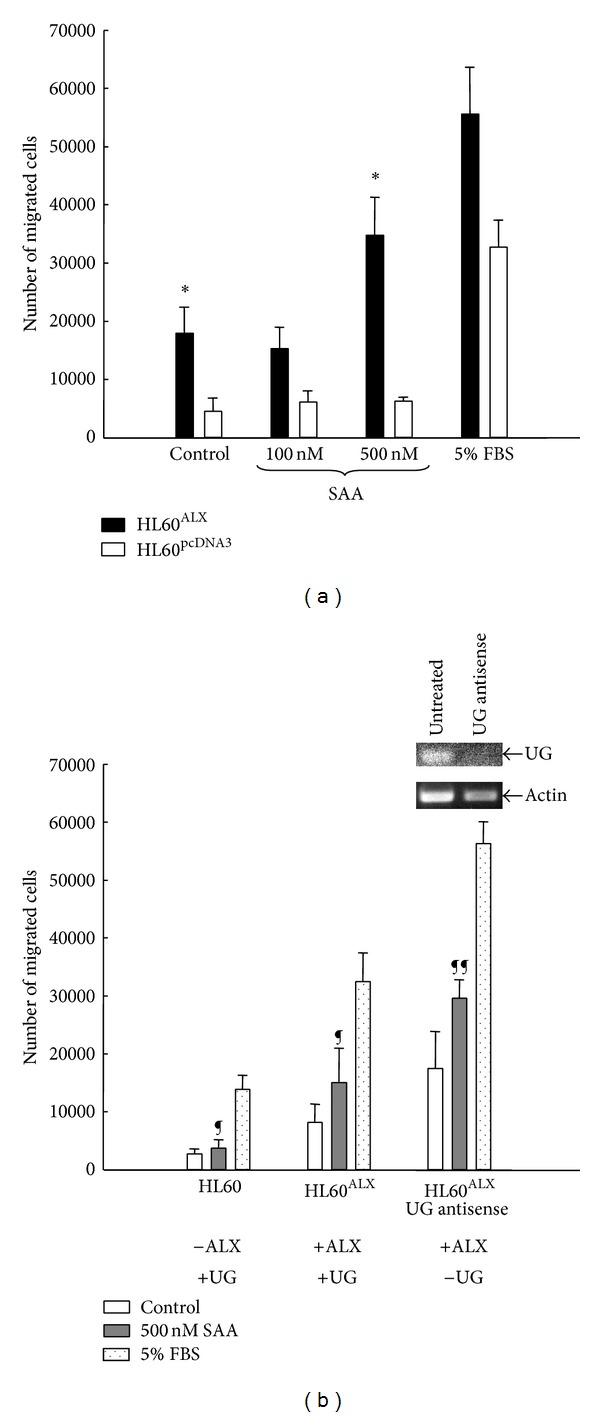
SAA-stimulated migration of HL-60 is mediated by ALX while being inhibited by UG. (a) Migration of HL60^ALX^ and HL60 into the lower chamber when SAA at two different concentrations (100 and 500 nM) or 5% FBS (positive control) are added. (b) Migration of HL60, HL60^ALX^, and UG-antisense treated HL60 cells when SAA (500 nM) or 5% FBS were added. Results are the mean ± SD of four separate experiments with triplicate determinations.

Chemotaxis is the primary consequence of the release of IL-8. Therefore, we determined the impact of UG on SAA-induced chemotaxis in both HL-60 cells and FLS. FLS are known to possess SAA-dependent ALX-mediated responses, including chemotaxis [7]. SAA had a dose-dependent effect on cell chemotaxis in HL-60^ALX^ (black bars) but not in mock transfected cells (white bars, Figure 4(a)). Its potency was compared to that observed with 5% FBS, a standard stimulus used to provoke chemotaxis via multiple, nonreceptor-restricted mechanisms, and also able to mobilize mock HL-60 cells (Figure 4). Besides the increase of chemotactic response caused by SAA, expression levels of ALX and endogenous UG were also found to correlate with the number of cells undergoing chemotaxis (Figure 4(b)). More specifically, baseline values in control samples more than doubled when comparing mock with HL-60^ALX^ cells and they increased even further if endogenous UG synthesis was silenced (Figure 4(b) and inset).

**Figure 5:**
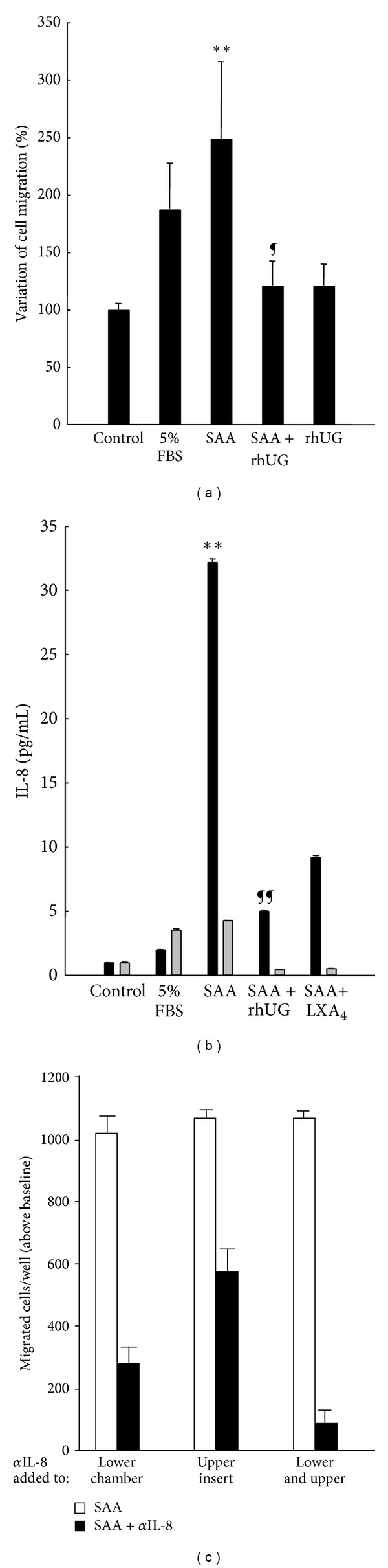
Attenuation of SAA induced cell migration and released IL-8 by hrUG in FLS. (a) Migration of FLS exposed to FBS, SAA (100 nM), or SAA with UG (100 nM), shown as % variation above control (basal growth medium). Results are the mean ± SD of three separate experiments with duplicate determinations. (b) Released IL-8 levels as measured by ELISA in the lower and upper chamber medium following FLS treatment with 5% FBS, SAA (100 nM) alone or in combination with UG or LXA_4_ (both at 100 nM). Results are the mean ± SD of duplicate determinations from two experiments. Data are normalized using cell numbers. (c) Migration of FLS following SAA (100 nM) treatment in lower chambers when IL-8 blocking antibody was added to lower, upper, or both compartments of the transwell system. Student's *t*-test SAA versus control *P* values of <0.005 (∗∗) and SAA versus SAA + UG treatments *P* values of <0.01 (¶) (a) and <0.002 (¶¶) (b). Results are the mean ± SD from quadruple determinations of two experiments performed.

The effects of SAA and UG on cellular chemotaxis were confirmed using human primary FLS, where only traces of decameric and higher molecular weight complexes of UG were visible as detected by western blots (slightly above the test detection limits of ~2 ng protein/3 × 10^6^ cells, data not shown). These UG amounts (calculated at ~12 pM) are negligible since they would represent concentrations of three log orders lower than the estimated affinity for ALX and ~1,000-fold less than that achieved with exogenously added UG (100 nM and Figure 1). Results in Figure 5 indicate that SAA-induced chemotaxis in human FLS is strongly inhibited by equimolar concentrations of UG, while UG alone had no effect on the number of migrated cells in control samples. In parallel experiments SAA caused a distinct elevation of IL-8 and determined a selective accumulation in the lower chamber compartment, while UG counteracted these effects (Figure 5(b)). Results also indicate that the SAA-induced IL-8 gradient is functionally relevant since its disruption with IL-8 blocking sera causes a significant reduction in the number of cells migrating from the upper to the lower compartment of the transwell (Figure 5(c)).

## 4. Discussion

Investigations with UG-knockout mice revealed that the absence of this protein can lead to phenotypes that suggest its critical homeostatic role/s in inflammation, autoimmunity, cancer, and other processes [37].

UG interactions with ALX seem to be linked to anti-inflammatory/proresolution events. While a growing number of ALX agonists have been reported, the characterization of their signaling pathways remains incomplete. In 2006, Kamal et al. reported that antiflammin-2 bound ALX, elicited postreceptor signaling responses, such as rapid phosphorylation of ERK1 and ERK2, and provided functional data in terms of inhibiting PMN interaction with endothelial monolayer under flow [29].

We report for the first time that UG could bind to ALX and through this receptor elicits inhibition of SAA-induced cellular PLA2 activity. UG binding to ALX yields a tonic inhibition of basal PLA_2_ activity levels. When being in combination with SAA, a rapid and pulsed PLA_2_ activation was observed, followed by inhibition of SAA activity. In fact, the PLA_2_ activation profile observed with SAA is typically observed with GPCRs activated by proinflammatory mediators such as FMLP or leukotriene B_4_ [38, 39]. UG pulsed PLA_2_ activation overlaps with the LXA_4_ profile that is accompanied by an extensive membrane lipid remodeling without the full activation of proinflammatory events, such as synthesis of proinflammatory eicosanoids from the unesterified AA [10, 12, 35]. Furthermore, the time profile of UG inhibition of SAA-induced PLA_2_ activity and the use of low concentrations of divalent cations in the assay (Ca^2+^ plus Mg^2+^ < 0.8 mM) strongly suggest that these events involve intracellular PLA_2_ isoforms, expanding on the UG repertoire of anti-inflammatory actions in addition to the inhibition of the secretory form of PLA_2_, as initially reported when UG properties were originally described [14].

Notably, further evidence of receptor antagonism between UG and SAA was obtained by measuring SAA-induced IL-8 release. The ALX role in UG and SAA signaling was established by suppressing its expression with ALX antisense oligo. The characterization of UG impact on this event was conducted using a HEC-1A, cell line devoid of endogenous UG synthesis, and stably transfected cells HEC-1A^UG^. Levels of IL-8 released after SAA challenge were found to be higher in HEC-1A than HEC-1A^UG^ and were affected by ALX antisense treatment. Notably, also the basal levels of IL-8 production were higher in wild type HEC-1A than in UG producing HEC-1A, suggesting that the autocrine action of UG is critical to both antagonizing SAA-induced, ALX-mediated release of IL-8, and regulating IL-8 basal levels. The role of endogenously synthesized UG on regulating basal level of IL-8 production overlaps with the inhibition of p38 phosphorylation basal levels and suggests that endogenous UG may be pivotal to “prevent” the ALX contribution to proinflammatory networks by opposing its association with proinflammatory ligands.

Ray et al. [40] reported that allergen exposure induces elevated expression of serum amyloid A in the lungs of UG knock-out mice. SAA higher production was found to provide a chemotactic signal for the migration of dendritic cells and UG was shown to inhibit SAA-induced migration of mL4-7 cells. These cells were generated by stably transfecting HEC-293 cells with FPR2 (a mouse homolog of the human FPRL1/ALX receptor) cDNA construct in a dose-dependent manner. In the current report, we also found that UG counteracts SAA-induced cell migration (as shown on nonadherent HL-60 cells in an ALX-dependent manner, as well as of adherent primary human FLS).

We have indication that UG synthesis occurs in a myeloid related cell lineage (data not shown). Our results indicate binding of *α*UG to HL-60^ALX^ with no further binding noted with the addition of exogenous UG. HL-60 cells were chosen for our studies since SAA-induced chemotaxis in myeloid cells and its dependence on ALX have been already established [6]. Our results obtained with HL-60 cells confirm that SAA is able to promote cell chemotaxis if cells are transfected with ALX. With use of UG antisense oligo to abolish endogenous UG synthesis, results indicate that loss of UG synthesis is accompanied by an exacerbated HL-60 cell chemotactic response to SAA. This suggested that, as proposed for IL-8 release in HEC-1A, endogenously synthesized UG exerts a tonic inhibition on activation of cell responses triggered by proinflammatory mediators.

While the use of engineered cell lines allowed for the intended characterization of UG interactions with ALX and resulting biological responses, FLS were used to evaluate the pathophysiological relevance of these events specifically in human synovium of arthritic joints. Moreover, the ability of cells to migrate upon SAA chemoattraction has been reported [6] so impact of UG on this chemotaxis was tested. UG was found to be a potent inhibitor of SAA-induced FLS chemotaxis and the inhibition was accompanied by a striking reduction in IL-8 levels. In fact, IL-8 measurements in the upper and lower chambers of the transwell system adopted for these experiments suggest that SAA, but not FBS, establishes a clear chemotactic gradient that is opposed by the presence of equimolar amounts of UG.

Taken together, there is a possibility that UG acts through pathways other than ALX. With the experiments performed we cannot rule out that fibronectin or other receptors might bind UG as well and trigger pathways, creating cross-talk signaling with ALX.

A single nucleotide polymorphism in the UG gene (A587G) appears to be associated with low risk of erosive disease in rheumatoid arthritis patients [41]. This indicates that UG could play a protective role in chronic inflammatory joint diseases. UG could be an attractive therapeutical option in the future, as reviewed by Pilon [42]. Evidence to this effect is coming from various fields of study. Mice lacking UG are highly susceptible to pulmonary fibrosis and recombinant UG was shown to prevent bleomycin-induced production of proinflammatory T-helper 2 cytokines and TGF-*β*, which are profibrotic. So, UG seems to be critical in preventing pulmonary fibrosis in mice [43]. Also, humans characterized to be deficient in UG exhibited tendencies toward inflammatory, fibrotic, and oncologic diseases [42]. UG was indicated to be likely to have a role as a lung surfactant-protective agent, especially important in neonatal respiratory distress syndrome. Human recombinant UG was tested in one of the first clinical trials for its effectiveness in protecting the surfactant, a complex mixture of phospholipids and proteins, from degradation by PLA2 catalysis and was shown to be safe when administered endotracheally [37]. UG was also discovered to prevent IgA mediated diseases, such as IgA nephropathy, by preventing the deposition of IgA-fibronectin immunocomplexes in tissues, such as the renal glomeruli [44].

In conclusion, identification of possible ALX-mediated UG activities extends the characterization of the anti-inflammatory properties previously described for UG. Our results indicate that UG can potently impact positive feedback mechanisms (IL-8 release) involved in SAA proinflammatory cell activation. UG may play a preeminent role in modulating inflammatory events across various target tissues. Its interaction with ALX, expressed on phagocytic cells, such as macrophages and PMN, as well as the currently documented ability of UG endogenous synthesis in cells of myeloid lineage, extends the pathophysiological relevance of UG anti-inflammatory activities beyond organs, such as lung and endometrium, where abundant presence of the protein and anti-inflammatory activities had been previously acknowledged [16, 45, 46].

## Abbreviations

ALX: Lipoxin A_4_ receptor
AP1: Activating protein 1
CHO: Chinese hamster ovary cells
FI: Fluorescence intensity
FLS: Fibroblast like synoviocytes
HEC-1A: Human endometrium cell line 1A
HL-60: Human promyelocytic cell line 60
IL-1*β*: Interleukin 1 beta
IL-8: Interleukin 8
LXA_4_: Lipoxin A_4_
NF-kB: Nuclear factor kappa B
PLA_2_: Phospholipase A_2_
SAA (rhSAA1): Serum amyloid A
UG (rhUG): Recombinant human uteroglobin, also known as CC10 protein.
